# A mixed-methods feasibility study protocol to assess the communication behaviours within the dental health professional-parent-child triad in a general dental practice setting

**DOI:** 10.1186/s40814-018-0331-3

**Published:** 2018-08-13

**Authors:** Siyang Yuan, Gerry Humphris, Al Ross, Lorna MacPherson, Yuefang Zhou, Ruth Freeman

**Affiliations:** 1 Dental Health Services Research Unit, School of Dentistry, University of Dundee, Park Place, Dundee, DD1 4HN UK; 2 Health Psychology, School of Medicine, University of St Andrews, St Andrews, UK; 3 School of Medicine, Dentistry, and Nursing, University of Glasgow, Glasgow, UK; 4 Public Health, NHS Tayside, Dundee, UK

**Keywords:** Triadic behaviour coding scheme, Children, Communication, Dental care, Video observation

## Abstract

**Background:**

The promotion of twice yearly application of fluoride varnish (FVA) to the teeth of pre-school children in the dental practice is one component of Scotland’s child oral health improvement programme (Childsmile). Nevertheless, evidence shows that application rates of FVA are variable and below optimal levels. The reasons are complex, with many contextual factors influencing activity. However, we propose that one possible reason may be related to the communication challenges when interacting with younger children. Therefore, the primary aim of the study is to assess the feasibility of conducting a video observational study in primary dental care. The secondary aim is to assess the communication behaviours of dental professionals and those of the parents to predict child cooperation when receiving FVA using this video observational study design.

**Methods:**

Approximately 50 eligible pairs of parents and child patients aged between 2 years and 5 years from general dental practices will be recruited to participate in the study. The consecutive mixed-method study will consist of two parts. The first part will be cross-sectional observations of the dental health professional-child-parent communication during dental appointments conducted in the general dental practice setting, using video recording. The second part will be a post-observation, semi-structured interview with parents and dental health professionals respectively. This will be implemented to explore their views on the acceptability and feasibility of being observed using video cameras during treatment provision.

**Discussion:**

The mixed-methods study will allow for directly observing the communication behaviours in the clinical setting and uncovering the views of participating dental health professionals and parents. Therefore, the study will enable us to [i] explore new ways to study the nature of triadic interaction of dental health professional-child-parent, [ii] identify dental health professionals’ effective communication behaviours that promote child patient and parent’s experience of using preventive dental service and [iii] to assess the feasibility of the study through uncovering the views of dental health professionals and parents.

## Background

Childsmile is a child oral health improvement programme in Scotland with components being delivered in the nursery, school, family home and dental practice settings [1]. It was introduced in 2006 due to the high prevalence of dental disease in pre-school children. Over the past 10 years the proportion of 5-year-old children in Scotland with no obvious decay experience has increased from 54% in 2006 to 69% in 2016 [2]. However, clear inequalities in dental health remain, with children from areas of high social deprivation still experiencing the majority of tooth decay. The programme continues to evolve to meet this challenge and in 2011 a payment system was introduced into the NHS Primary Care payment system, whereby dental practitioners are now remunerated for the twice yearly application of fluoride varnish to the teeth of children aged between 2 and 5 years. This was informed by the evidence base on the effectiveness of fluoride varnish in preventing childhood caries [3]. Thus, Childsmile promotes the use of twice yearly fluoride varnish application (FVA), along with oral health education (e.g. fluoride toothpaste use; dietary counselling and advice) for preschool children in general dental practice. However, despite practices being financially remunerated, FVA provision remains low, with national monitoring data for 2015/16 showing that only 18% of 2–5 year old children registered with a dental practitioner in Scotland receiving the recommended two applications of fluoride varnish within a year [4]. Recent work has shown that the reasons are complex, with contextual factors including the interaction between the dental health professional, parent and child [5]. It is therefore important to explore the communication behaviours within the dental health professional-parent-child triad during the dental treatment session and to examine how communication behaviour affects successful FVA in primary dental care.

The current literature provides some answers. It suggests that children’s behaviours and anxiety when undergoing dental-related treatments can affect successful treatment outcomes [6–10]. However, little research, if any, has systematically focused on the triadic interaction and the impact of dental health professionals’ communication behaviours on child patients and their parents. Studies have predominantly focused on the dyadic interactions between doctors/dentists and their adult patients [11–14]. These previous studies in the dental setting used video recordings to explore communication behaviours in general dental practices with regard to patient dental anxiety [15, 16]. The only work that has examined intensively dental health professionals and child patient communication is the BEHAVE study, which took place in the nursery school setting [17, 18]. The BEHAVE study demonstrated the value of observing directly, using video recording, the interaction between two extended duty dental nurses (EDDNs) (who applied the varnish) and the child receiving the fluoride application in the Childsmile Nursery programme [17]. Findings of the BEHAVE Study suggested that EDDNs were effective in predicting children’s anxiety and showed the appropriate supportive behaviour (‘preparation time’) prior to successful FVA [18]. Moreover, the BEHAVE study showed that the presence of the camera and researcher did not affect EDDNs’ communication behaviours, FVA, nor did it cause distress to EDDNs or child participants [19]. Therefore, while BEHAVE observed a triadic communication between 2 EDDNs and the child, there is an absence of research using video recording to examine the triadic interaction between dental health professionals, child patients and their parents in the clinical setting of general dental practice.

**Fig. 1:**
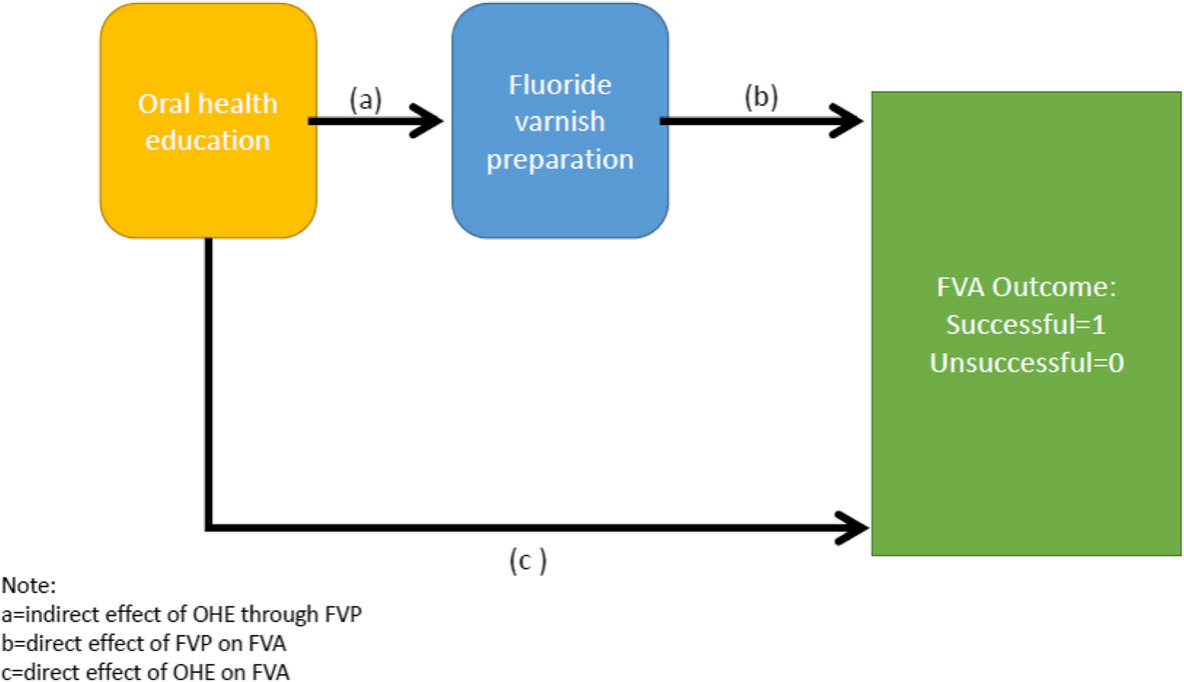
Hypothesised mediation model for predicting FVA outcome

**Fig. 2:**
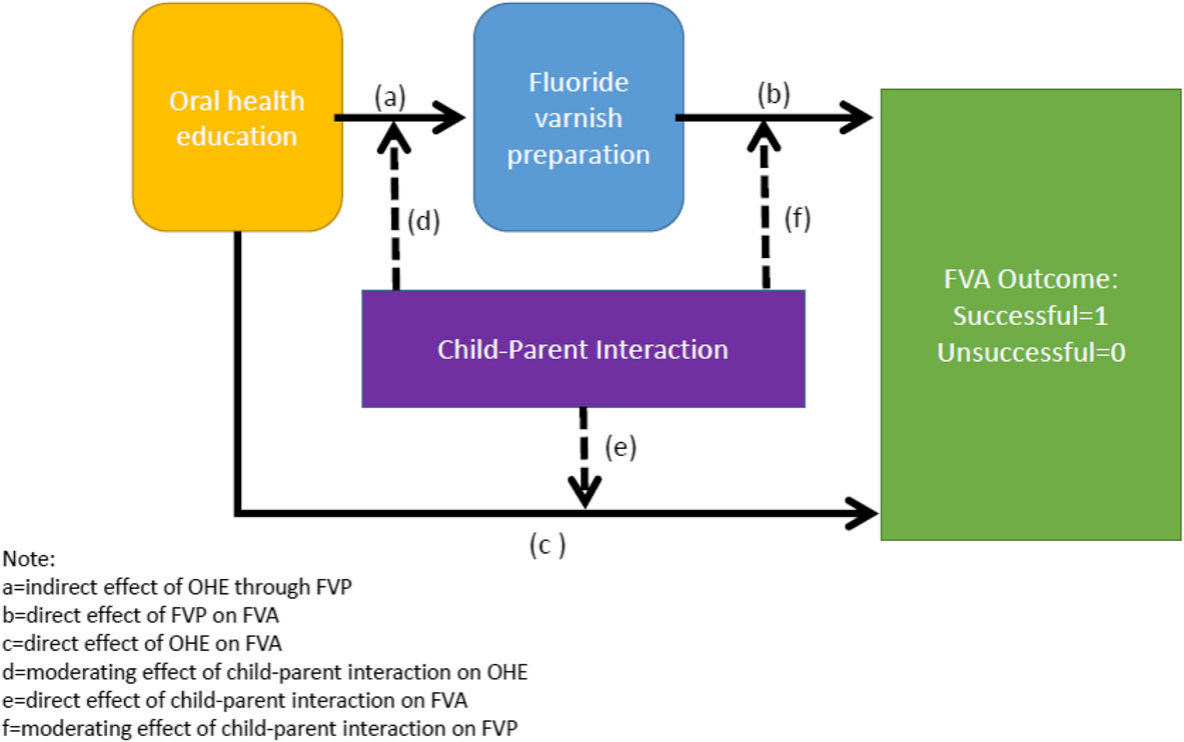
Hypothesised moderation/mediation model for predicting FVA outcome including child-parent interaction

Childsmile practice, therefore, presents an interesting new set of challenges if preschool children are to receive FVA successfully. The important issue for the present study is that it is unclear what the role of oral health education for child patients and their parents is, with regard to FVA—for instance could the dental health professionals’ communication behaviours during OHE prepare the child or improve successful FVA in preschool children? In essence, what is proposed here is the use of a moderator–mediator modelling approach to tease out the direct and indirect influences of dental health professional communication on FVA outcome. Hence, the dental health professional communication behaviours during oral health education mediate the staff behaviour during the FVA preparation time, whereas the child-parent interaction acts as a potential moderator, for successful FVA for the preschool child (Figs. 1 and 2). This study therefore examines the communication behaviours of dental health professionals with parent and/or child, as well as those of the parent-child, when providing oral health education and preparing the child patient for FVA as predictors of successful fluoride varnish application.

### Aims of study

The primary aim of the study is to assess the feasibility of conducting a video observational study in primary dental care. The secondary aim is to assess the communication behaviours of dental professionals and those of parents to predict child cooperation when receiving FVA using this video observational study design. The primary objectives are to:

1. Assess the feasibility (recruitment) of collecting video recordings of Childsmile dental consultations in the general dental practice.
2. Explore dental professionals’ and parents’ views relating to feasibility, acceptability and practicalities.
3. Assess the feasibility of developing a communication coding scheme to measure the communication between dental professionals, parents and children.

The secondary objectives are to:

1. Identify the communication behaviours of dental health professionals that predict successful FVA.
2. Investigate whether the child-parent communication acts as a moderator to successful FVA and in addition examine if oral health education provision by the dental health professional acts as a preparation to FVA and contribute to successful FVA.

## Methods/design

### Setting

In Scotland, all practices delivering NHS care to children are expected to deliver the Childsmile interventions outlined above. The setting of the present study will be NHS general dental practices in two NHS boards located in the East of Scotland. A child dental appointment in the NHS general dental practice will include:

[i] Dental check-up (if the appointment is with a dentist).
[ii] Oral health education (OHE) including fluoride toothpaste use, dietary counselling and oral hygiene advice.
[iii] FVA with aftercare advice as appropriate.

### Study design

The study design will be a consecutive mixed-methods study [20]. This will consist of two parts:

[i] A series of cross-sectional observations of the dental health professional-parent-child interaction during fluoride varnish application and/or oral health education provision will be conducted in a general dental practice setting using video recording.
[ii] A post-observation, semi-structured interview with parents and dental health professionals, separately, will explore their views and opinions on the acceptability and feasibility of being observed using video cameras during treatment provision.

### Participants

It is estimated that at least four general dental practices are required with a sample of 50 child-parent dyads with around 12–13 dyads per practice. It is regarded neither appropriate nor necessary to complete a detailed sample size calculation for the present feasibility study [21].

#### Inclusion criteria

All dental health professionals (dentists, dental therapists, dental hygienists and extended duty dental nurses under the supervision of dentists) working in NHS general dental practices in the two NHS health board regions and providing written consent will be identified as potential participating dental health professionals.

All children aged between 2 and 5 years who attend general dental practices in the selected regions for OHE and FVA (and/or a dental check-up) with their parents and providing written consent will be identified as potential child and parent participants.

#### Exclusion criteria

Dental health professionals who are not trained and eligible to provide FVAs on children and those who do not work in the two selected NHS boards in the East of Scotland and those not providing written consent will be excluded from this study.

Children aged less than 2 years or more than 5 years, children with learning disability, those families with little knowledge of English and who require an interpreter and those not providing written consent will be excluded from the study.

### Materials and procedure

#### Recruitment of participants

##### General dental practices/professionals

NHS general dental practices will be recruited using a combination of the following methods:

[i] The research team have already established good links with four general dental practices within two NHS boards which have expressed an interest, in principle, in taking part in a video study.
[ii] Information will be sent by mail to all NHS general dental practices in the selected areas of the two NHS boards, and interested practices will be visited by the researcher (SY).
[iii] As a registered research project, the present study will recruit potential dental practitioners in the two NHS board areas through the Scottish Dental Practice Based Research Network.
[iv] A snowball recruitment method will also be used with dental health professionals engaged in the study recommending other colleagues who might be interested in participating in the research [22].

##### Child-parent dyads

Dental staff will initially invite parents and their children to participate. Parents will be approached through the dental health professionals in the participating general dental practices. Once a dental appointment with a child aged between 2 and 5 years for FVA is made, the participating dental health professionals will send the participating information sheet to the parent through the post and contact the researcher (SY) about the date and time of the appointment. The researcher will attend the practice on the day of the appointment and will explain to the potential participating parents the aim and content of the study and invite them to take part. An information sheet will be given and fully informed parental consent will be required before participation.

### Sample size

As this is a feasibility study, a formal sample size calculation will not be required [23]. We plan to recruit four general dental practices with a total sample of 50 child-parent dyads. This will be a large enough sample to inform feasibility and acceptability of conducting the video observational study.

#### Video recording of the observations on the interactions

Observations of the triadic dental health professional-parent-child communication and interactions during the dental appointment for OHE and FVA will take place in general dental practice in the form of video recording. From the BEHAVE study [18], the FVA, on average, takes 5 min; however, as the present feasibility study will occur in the general dental practice setting, it is expected that the children may have a dental examination, with oral health education provision, in addition to fluoride varnish application. Therefore, the session will include not only the FVA but also the communication behaviours between dental health professional, parent and child concerning OHE. This is estimated to take between 5 and 20 min. We postulate that there will be substantial variation. This is an essential feature of the feasibility study to enable estimates of time expended on these interaction sessions.

A small-sized digital camera (Canon HD Camcorder LEGRIA HF R76) will be used and placed approximately 2 m on the tripod from the dental health professionals, parent and child. The operation of the recording system will be well practised, and the setup will be completed in good time to minimise any possible disturbance to the dental health professionals, parents and children. Another camera (Canon HD Camcorder LEGRIA HF R76) will be hand-held to pick up the key features of the interaction and/or capture any communication behaviours or interaction missed from the video observations due to the surgery layout.

The recording and storage of video files will strictly follow extensive ethical procedures. They include the sitting of the material in a swipe card and key pad security-entry coding room. The protocol for this facility is well established and includes, for example, the provision of non-networked PC computers, audit trail of file storage, and on-line training (MRC) of research team members on the use of confidential clinical data. Other features include the digital obscuring of the face images of relevant participants and using the obligatory data file storage in a locked cabinet. In addition, the digitalised files on the computer will be permanently removed from the computer once the coding and analysis are complete. They will be exported, after face images have been digitally blurred, onto an external hard drive that will have all data encrypted, and access to data files will be password protected. These encrypted data will be stored for 5 years following the end of the study, and the original raw data on DVDs will be permanently destroyed at the end of the project.

#### Exit interviews with dental health professionals and parents

Exit interviews with dental health professionals and parents (on an individual consent basis) will briefly ask about their perceived acceptability about the process (i.e. using two video cameras to observe the consultation in a dental setting). Interviews are estimated to take up to 15 min (Table 1).

**Table 1:** Exit semi-structured interview items

Dental health professionals questions	Parents questions
1. What are your general reflections on the video recording of your CS activity? 1.1 What went well? 1.2 What if anything was a problem? How could this be overcome, if at all?	1. How did you find being video recorded? 1.1 Were there any bad things? How could these be overcome? 1.2 What about any benefits?
2. How, if at all, did the recording affect your ‘normal’ Childsmile practice? 2.1 What could we do to make this as naturalistic as possible?	2. Did it feel like a ‘normal’ experience at the dentist?
3. What would be your key recommendation (s) if this was taken forward in a bigger study? 3.1 What further resources, if any, would practitioners need?	3. Is there anything else you wish to tell us about?
4. Is there anything else you wish to tell us about?	

#### Follow-up interviews with dental health professionals

The dental health professionals (at least one from each practice) will be contacted again for an in-depth follow-up interview after video data collection is completed with the practice. The interview will invite dental health professionals to discuss their views about the feasibility, practicality and acceptability of the format of this study that uses video cameras to observe their clinical practice of FVA on children with the presence of the child’s parent. Discussion will also include explorations on themes from exit interviews, general perceptions of the organisation of the study and understandings and views of FVA in general dental practice (Table 2).

**Table 2:** Follow-up interview items with Childsmile dental health professionals

1. What did you think about the video recording of your Childsmile activity? 1.1. What do you think went well? 1.2. What if anything do you think was a problem or caused a problem for your daily routines in practice? 1.2.1. How do you think this could this be overcome, if at all? 2. [Feed in here themes from exit analysis- “other dentists and patients have reported … …”] 2.1. Do you agree or disagree these issues could be barriers/facilitators? 2.2. What suggestions would you make for each? 3. How, if at all, did the recording affect your “normal” Childsmile Practice? 3.1.What could we do to make the videoing procedure more naturalistic? 4. Recommendations: 4.1 What would be your key recommendation (s) if this was work taken forward in a bigger study? 4.2. What further resources, if any, would you need participate? 5. Do you think it would be helpful and useful to have feedback sessions with practitioners like yourself, after we have completed the video analysis and review? 7. Is there anything else you wish to tell us?	

### Outcome measures

The outcomes are split into primary outcomes (i.e. feasibility) and secondary outcomes (i.e. clinical outcome).

Therefore, the primary outcome measures are as follows:

1. The recruitment of 50 child-parent dyads from 4 NHS general dental practices and collection of 50 dental professional-child-parent Childsmile dental consultations.
2. Dental professionals and parents’ views can be compiled relating to feasibility, acceptability and practicalities.
3. A communication coding scheme can be produced to measure the communication interactions between dental professionals, parents and children.

The secondary outcome measures are as follows:

1. The frequency or duration of communication behaviours of dental health professionals identified during the oral health education session predict successful FVA.
2. The frequency or duration of child-parent communication behaviours that act as a moderator to successful FVA and the frequency or duration of the dental health professionals’ oral health education-related communication behaviours that act as a preparation to FVA and contribute to successful FVA.

### Data analysis

#### Video data coding and analysis

The video will be transformed using specialist software (MOVAVI) for placing into suitable format (MPEG-4) for entry into bespoke Noldus software. All video data will be analysed using the Observer XT 10.5 system. The Observer XT 10.5 is a behaviour software package to facilitate coding, management and analysis of observational data. This software allows for the linking of particular behaviours (e.g. oral health information giving behaviour) to the subject (e.g. dental professional) who initiated the behaviour. First, a coding scheme will be developed based on the verbal and non-verbal behaviours of dental health professionals, children and their parents from collected video data. A general principle in our design has been the helpful guide produced by Chorney et al. [24] for the development and modification of paediatric behaviour coding schemes. As the focus of the present study is to understand how dental professionals and parents’ behaviours encourage children’s participation in the consultation and their cooperation in receiving FVA, the coding scheme will be designed therefore to be sensitive enough to detect child’s responses to dental professional and parental behaviours. We will develop a new coding scheme that not only focuses on dental professionals’ encouragement and communication strategy and children’s responses but also on the mechanism of [1] whether or not dental professionals’ interactive behaviours can motivate child participation in oral health education and prepare them for receiving FVA and [2] whether or not the child-parent interaction can serve as a moderator to child’s cooperation of receiving FVA. Therefore, a communication behaviour coding scheme will be developed based on the St Andrews Behavioural Interaction Coding Scheme (SABICS) which was designed to record interactive behaviours between dental nurses and 3–5 year-old children in a nursery setting during the fluoride varnish application sessions of Childsmile Programme [25]. The new coding scheme will include additional codes to record the interactive behaviours of dental professionals, child and parent in oral health education and child-parent interaction throughout the whole consultation in a general dental practice setting. The audio-visual recordings (video) will be coded using:

[i] Modified SABICS to code the FVA session.
[ii] A new set of coding categories to capture the elements of oral health education.
[iii] A new set of coding categories to assess child-parent interaction during the consultation.

Cohen’s Kappa with 95% confidence intervals will be applied to check both inter- and intra-coder reliability by conducting test-retest on 10% of video tapes for the entire coding scheme. We will check agreement on the following:

[i] Whether a particular behaviour takes place.
[ii] Whether behaviours happened at a same time. The tolerance window will be set to 1 s.

The following essential analyses will be conducted. The frequency or duration of certain behaviours of the dental health professionals, parents and children will be calculated to identify possible relationships between these behaviours and FVA outcome. This final step (mixed modelling––see below) will be dependent on the final number of videos collected, the frequency of certain key behaviours and from the initial univariate exploration of frequency distribution of behaviours. Multiple high effect sizes will enable a multivariate analysis similar to the type previously performed with the BEHAVE study [18]. As explained below, the analysis strategy of the BEHAVE study will be extended to incorporate the increased complexity of the GDP setting. The power of such analyses will be relatively low however with only 50 interactions to investigate; hence, this part of the project will be dependent on the usability and quality of the videos and complimentary audio. Such analyses will be of great assistance in developing future proposals to obtain higher levels of statistical power. Therefore, for the present study, emphasis will be placed on effect estimation and confidence intervals rather than *p* values as the study has not been powered to detect differences [26, 27].

The secondary (clinical) outcome measure will therefore be whether or not there is successful fluoride varnish application for the preschool child. A mixed structural equation model with binomial link function to the dependent variable (FVA application) will be conducted. An important question for the investigation is to determine whether suitable models as presented in Figs. 1 and 2 can be prepared and preliminarily tested with a relatively small data set. The flexibility of mixed linear modelling using the feature of full information maximum likelihood available in MPlus will be employed to assist prediction of successful outcomes of FVA with the child patients. This study will provide valuable evidence of the variability of the dental staff and parental-child behaviour to ascertain whether there are direct/indirect mediating/moderating effects between variables of oral health education, fluoride varnish preparation and/or child-parent interaction on predicting the outcome of successful FVA as outlined in the two hypothesised models (Figs. 1 and 2).

#### Interview data analysis

Interview notes and any supplementary observations made during the feasibility study will be written up as soon as practical to computer files. The interview notes will be transcribed fully and again transferred to electronic files for analysis.

The interview and direct observation data will be coded and organised into broad themes using thematic analysis techniques [28] and facilitated by QSR NVivo 10.0 qualitative analysis software. The qualitative data will be examined for themes by SY. Each of the emerging themes will be reviewed independently by other members of the research team to ensure data coherence, that the theme aptly summarises the data and trustworthiness. This process will be repeated several times until the themes identified are coherent, exhaustive and informative.

### Ethical considerations

Ethical approval for the study has been granted from the East of Scotland Research Ethics Service (REC reference 16/ES/0081) and NHS R&D Management Approvals from two NHS Boards (NRS reference NRS16/188980) have been granted.

## Discussion

### Importance of the project

Improving oral health and reducing oral health inequality in childhood is of central importance in Scotland. The Childsmile Programme has shown great success; however, children from the most deprived areas still have the greatest experience of obvious decay experience. Despite high-quality evidence of the benefits of FVA, the proportion of children receiving this preventive intervention remains low. We have proposed that one possible reason for this situation may be related to the communication challenges when interacting with younger children; thus, we have suggested that it is essential to examine how dental health professionals communicate within a triadic interaction involving the parent, child and dental health professional to ensure successful FVA.

This study will collect some initial data to populate two models (Figs. 1 and 2). The rationale of both models is to translate the objectives into a testable approach to examine the relationships hypothesised. The data collected from the observations catalogued into Observer XT into a formatted file for entry into MPlus requires transformation and initial testing for identification and potential to return estimates of model fit. The first model presents a simplified causal model with oral health education specified as a distal variable to explaining FVA success mediated by the proximal variable FVA preparation. The second model presents the same configuration but with the moderating influence of the child-parent interaction which is hypothesised to be central to the success of the dental visit.

To our knowledge, there are no previous studies investigating the triadic communication interactions between dental health professionals, parents and young children using video recordings in the primary dental care setting. Moreover, little is known about the communication behaviours that the dental health professionals use when delivering OHE to parents and children and whether this influences successful FVA outcomes, within the session or subsequent FVA sessions. The present study will provide insights to disentangle the complexities (mediation or moderation) of communication behaviours in such triadic encounters in the primary dental care setting. This project will thus provide essential evidence to plan additional studies to better understand the procedures that provide for successful FVA in preschool children.

### Limitations

The presence of a researcher and two digital video cameras may disrupt the routines and usual practice in the dental surgery. However, evidence from the BEHAVE study suggests that the presence of the camera and the researcher will not have any measurable effect on the dental health professionals’ delivery of the varnish application, nor is this likely to upset children or parents [19].

Those dental health professionals who agree to participate might be those who are either confident with their effective communication skills or have previous experience of being video recorded. This may cause selection bias which result in poor representation of the dental health professional sample and associated communication behaviours. Similarly for parents and children, those who agreed to take part in the study might be those with less dental anxious behaviours and more favourable experiences in the healthcare settings. However, this will be countered by including first-time children and their parents to Childsmile practice for OHE and FVA.

In summary, this protocol is to explore the communication behaviours within the dental health professional-parent-child triad. The aim is to assess the feasibility of conducting a video observational study in primary dental care and to assess the communication behaviours of dental professionals and those of parents to predict child cooperation when receiving FVA using this video observational study design. In order to achieve this aim, a mixed-methods research design will be used, involving video recording (quantitative) and post-observation interviews (qualitative) with both parents and dental health professionals. Doing so will allow for directly observing the communication behaviours in the clinical setting and uncovering the views and opinions of participating dental health professionals and parents. Therefore, this study will allow us to:

1. explore new ways to study the nature of triadic interaction of dental health professional-child-parent;
2. identify dental health professionals’ effective communication behaviours that promote child patient and parent’s experience of using the preventive dental service;
3. explore the views of dental health professionals and parents with regard to feasibility of conduct audiovisual observations in the general dental practice setting.

## Conclusion

The present mixed-methods study will enable us to [i] use a detailed schedule of procedures to guide the observational research and to establish a testable model and [ii] also to explore the views of dental health professionals and parents with regard to feasibility of conduct audio-visual observations in the general dental practice setting.

## Abbreviations

EDDN: Extended duty dental nurse
FVA: Fluoride varnish application
GDP: General dental practice
MRC: Medical Research Council
NHS: National Health Service
OHE: Oral health education
